# Supplementary material for: Combining DNA Barcoding and HPLC Fingerprints to Trace Species of an Important Traditional Chinese Medicine Fritillariae Bulbus
Source: Molecules. 2019 Sep 8;24(18):3269. doi: 10.3390/molecules24183269 (PMC6766824; doi:10.3390/molecules24183269)
Supplement: Supplementary file 1 [file molecules-24-03269-s001.zip › Supplement/Supplementary Table/TableS1.docx]

Table S1 the information of samples

| Species | | No | Locality | ITS | ITS2 | deposited |
| --- | --- | --- | --- | --- | --- | --- |
| *Fritillaria cirrhosa D.Don* | | J8 | qingchuan,sichuan | 611 | 238 | 1 |
|  | | J24 | luhuo,sichuan | 611 | 238 | 1 |
|  | | J29 | shenyang,liaoning | 611 | 238 | 1 |
|  | | J38 | xiaojin,sichuan | 611 | 238 | 1 |
|  | | J46 | ganzi,sichuan | 611 | 238 | 2 |
| *Fritillaria taipaiensis P.Y.Li* | | T5 | hongciba,chongqing | 611 | 238 | 1 |
| *Fritillaria delavayi Franch* | | S4 | ganzi,sichuan | 610 | 238 | 2 |
|  | | S7 | qizhou,hebei | 610 | 238 | 2 |
|  | | S11 | ganzi,sichuan | 610 | 238 | 2 |
|  | | S15 | ganzi,sichuan | 610 | 238 | 2 |
|  | | S20 | xiaojin,sichuan | 610 | 238 | 2 |
|  | | S21 | guoluo,qinghai | 610 | 238 | 1 |
| *Fritillaria przewalskii Maxim* | | G2 | weiyuan,gansu | 611 | 238 | 1 |
|  | | G4 | min,gansu | 611 | 238 | 1 |
|  | | G7 | jinzhong,gansu | 611 | 238 | 2 |
|  | | G8 | wudu,gansu | 611 | 238 | 2 |
|  | | G9 | yaojiansuo,gansu | 611 | 238 | 2 |
| *Fritillaria unibracteata Hsiao et K.C.Hsia* | | A4 | pansong,sichuan | 611 | 238 | 1 |
|  | | A11 | hengtai,guangdong | 611 | 238 | 1 |
|  | | A14 | ganzi,sichuan | 611 | 238 | 1 |
|  | | A16 | ruoergai,sichuan | 611 | 238 | 1 |
|  | | A17 | aba,sichuan | 611 | 238 | 2 |
| *Fritillaria walujewii Regel* | | X1 | yili,xinjiang | 628 | 239 | 2 |
|  | | X3 | lasa,xizang | 628 | 239 | 2 |
|  | | X6 | lianqiao,hunan | 628 | 239 | 1 |
|  | | X12 | yili,xinjiang | 628 | 239 | 1 |
| *Fritillaria ussuriensis Maxim* | | P3 | jiulong,sichuan | 742 | 235 | 2 |
|  | | P4 | qizhou,hebei | 742 | 235 | 2 |
|  | | P5 | qingyuan,liaoning | 742 | 235 | 1 |
|  | | P6 | liaoyang/liaoning | 742 | 235 | 1 |
|  | | P7 | tonghua,jilin | 742 | 235 | 1 |
| *Fritillaria thunbergii* | | Z4 | panan,zhejiang | 609 | 238 | 2 |
|  | | Z5 | baoding,hebei | 609 | 238 | 2 |
|  | | Z7 | panan,zhejiang | 609 | 238 | 2 |
|  | | Z8 | dongyang.zejiang | 609 | 238 | 1 |
|  | | Z13 | panan,zhejiang | 609 | 238 | 1 |
| *Fritillaria pallidiflora Schrenk* | | Y1 | yili,xinjiang | 628 | 239 | 1 |
|  | | Y2 | yili,xinjiang | 628 | 239 | 1 |
|  | | Y3 | yili,xinjiang | 628 | 239 | 2 |
|  | | Y4 | yili,xinjiang | 628 | 239 | 2 |
|  | | Y5 | yili,xinjiang | 628 | 239 | 2 |
| *Fritillaria hupehensis Hsiao* | | H2 | wuxi,chongqing | 780 | 238 | 2 |
|  | | H3 | wuxi,chongqing | 780 | 238 | 2 |
|  | | H5 | wuxi,chongqing | 780 | 238 | 2 |
|  |  |  |  |  |  |  |

1 Medicinal materials library, School of Chinese Materia Medica, Tianjin University of Traditional Chinese Medicine

2 Herbarium, Institute of Medicinal Plant Development, Chinese Academy of Medical Sciences & Peking Union Medical College
