# Supplementary material for: Combining DNA Barcoding and HPLC Fingerprints to Trace Species of an Important Traditional Chinese Medicine Fritillariae Bulbus
Source: Molecules. 2019 Sep 8;24(18):3269. doi: 10.3390/molecules24183269 (PMC6766824; doi:10.3390/molecules24183269)
Supplement: Supplementary file 1 [file molecules-24-03269-s001.zip › Supplement/Supplementary Table/TableS2.docx]

Table S2 Accession Number of GenBank

| NO | Accession Number ITS | Accession Number ITS2 |
| --- | --- | --- |
| J8 | MN184744 | MN184801 |
| J24 | MN184746 | mn184799 |
| J29 | MN184754 | MN184796 |
| J38 | MN184750 | MN184798 |
| J46 | MN184743 | MN184797 |
| T5 | MN184768 | MN184824 |
| S4 | MN184753 | MN184802 |
| S7 | MN184748 | MN184800 |
| S11 | MN184747 | MN184803 |
| S15 | MN184745 | MN184807 |
| S20 | MN184751 | MN184804 |
| S21 | MN184756 | MN184806 |
| G2 | MN184765 | MN184820 |
| G4 | MN184764 | MN184821 |
| G7 | MN184767 | MN184822 |
| G8 | MN184769 | MN184826 |
| G9 | MN184763 | MN184818 |
| A4 | MN184777 | MN184835 |
| A11 | MN184778 | MN184830 |
| A14 | MN184781 | MN184842 |
| A16 | MN184774 | MN184843 |
| A17 | MN184775 | MN184837 |
| X1 | MN184787 | MN184831 |
| X3 | MN184786 | MN184834 |
| X6 | MN184782 | MN184836 |
| X12 | MN184788 | MN184839 |
| P3 | MN184772 | MN184838 |
| P4 | MN184779 | MN184840 |
| P5 | MN184783 | MN184841 |
| P6 | MN184780 | MN184833 |
| P7 | MN184784 | MN184832 |
| Z4 | MN184766 | MN184827 |
| Z5 | MN184770 | MN184825 |
| Z7 | MN184776 | MN184823 |
| Z8 | MN184773 | MN184828 |
| Z13 | MN184771 | MN184829 |
| Y1 | MN184758 | MN184815 |
| Y2 | MN184757 | MN184810 |
| Y3 | MN184761 | MN184817 |
| Y4 | MN184762 | MN184819 |
| Y5 | MN184760 | MN184816 |
| H2 | MN184752 | MN184809 |
| H3 | MN184759 | MN184805 |
| H5 | MN184755 | MN184808 |
