# Supplementary material for: Combining DNA Barcoding and HPLC Fingerprints to Trace Species of an Important Traditional Chinese Medicine Fritillariae Bulbus
Source: Molecules. 2019 Sep 8;24(18):3269. doi: 10.3390/molecules24183269 (PMC6766824; doi:10.3390/molecules24183269)
Supplement: Supplementary file 1 [file molecules-24-03269-s001.zip › Supplement/Supplementary Table/TableS3.docx]

Table S3 Content of main alkaloid in Fritillariae Bulbus

| Sample | Peimisine | Verticine | Verticinone | Imperialine |
| --- | --- | --- | --- | --- |
| J8 | 101.20 | 70.10 | 27.20 | 34.20 |
| J24 | 117.10 | nd | 68.90 | 77.20 |
| J29 | 36.10 | 61.30 | nd | nd |
| J46 | 41.50 | nd | 22.50 | 45.30 |
| J38 | 206.30 | nd | 39.40 | 55.10 |
| T5 | 278.20 | 46.60 | 42.20 | 27.90 |
| A4 | 57.50 | 69.70 | 48.70 | 33.90 |
| A11 | 56.20 | 92.80 | 33.00 | 36.70 |
| A14 | 49.90 | 102.10 | 29.10 | 34.80 |
| A16 | 69.80 | 93.70 | nd | 33.60 |
| A17 | 65.30 | 98.70 | nd | 37.70 |
| S4 | 30.00 | 73.90 | nd | nd |
| S7 | 47.60 | 63.70 | 91.40 | 58.40 |
| S11 | nd | 78.10 | nd | nd |
| S15 | 42.90 | 31.60 | nd | 49.40 |
| S21 | 28.30 | nd | nd | 37.30 |
| S20 | 57.40 | 116.50 | 53.40 | 36.20 |
| G2 | 140.70 | 28.70 | 54.10 | 153.20 |
| G4 | 180.50 | 39.60 | nd | 39.90 |
| G7 | 256.90 | 45.10 | 75.70 | 42.60 |
| G8 | 294.40 | 51.20 | nd | 46.70 |
| G9 | 262.20 | 38.80 | 41.80 | 32.20 |
| Z4 | 159.80 | nd | 588.10 | 691.00 |
| Z5 | 27.70 | nd | 577.30 | 705.60 |
| Z7 | 106.00 | nd | 734.20 | 855.60 |
| Z8 | nd | nd | 426.60 | 557.50 |
| Z13 | 85.10 | nd | 482.20 | 635.00 |
| Y1 | 58.70 | 328.00 | nd | nd |
| Y2 | 177.90 | 756.20 | nd | nd |
| Y3 | 146.20 | 647.80 | nd | nd |
| Y4 | 56.50 | 657.90 | nd | 179.50 |
| Y5 | 104.00 | 616.00 | nd | nd |
| X1 | 129.50 | 212.20 | nd | nd |
| X3 | 112.70 | 272.80 | nd | nd |
| X6 | 401.10 | 442.90 | nd | 102.90 |
| P3 | 97.10 | 45.30 | 43.90 | nd |
| X12 | 219.60 | 236.60 | nd | 128.80 |
| H3 | 668.50 | nd | 1018.90 | 152.60 |
| H2 | 547.40 | nd | 919.30 | 399.40 |
| H5 | 975.20 | nd | 1095.20 | 401.60 |
| P4 | 94.90 | 47.40 | 47.80 | 34.40 |
| P5 | 97.10 | 32.50 | 25.80 | nd |
| P6 | 94.90 | nd | 47.80 | 34.40 |
| P7 | 147.90 | 88.90 | 54.40 | 69.40 |

nd: not detected
