# Supplementary figures and images for: Combining DNA Barcoding and HPLC Fingerprints to Trace Species of an Important Traditional Chinese Medicine Fritillariae Bulbus
Source: Molecules. 2019 Sep 8;24(18):3269. doi: 10.3390/molecules24183269 (PMC6766824; doi:10.3390/molecules24183269)

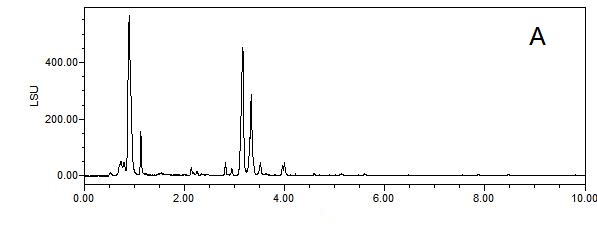

Supplement: Supplementary file 1 [file molecules-24-03269-s001.zip › Supplement/Supplementary Figure/FigS1.jpg]

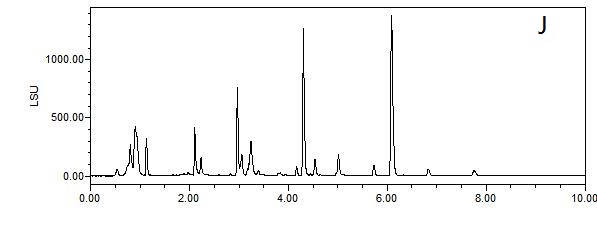

Supplement: Supplementary file 1 [file molecules-24-03269-s001.zip › Supplement/Supplementary Figure/FigS10.jpg]

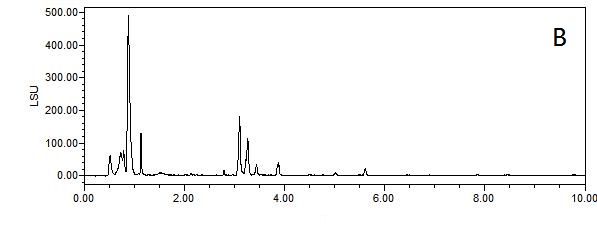

Supplement: Supplementary file 1 [file molecules-24-03269-s001.zip › Supplement/Supplementary Figure/FigS2.jpg]

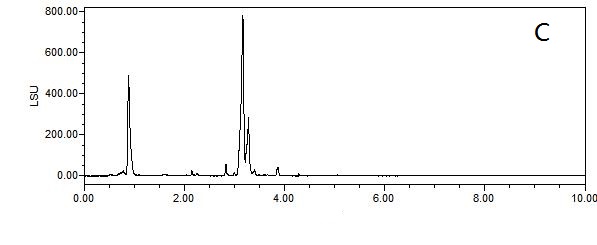

Supplement: Supplementary file 1 [file molecules-24-03269-s001.zip › Supplement/Supplementary Figure/FigS3.jpg]

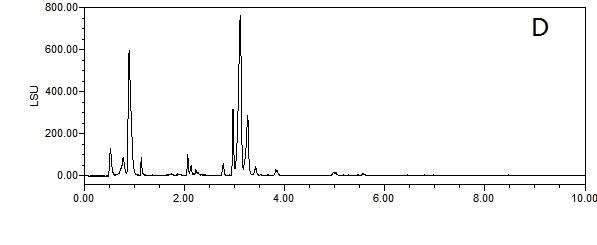

Supplement: Supplementary file 1 [file molecules-24-03269-s001.zip › Supplement/Supplementary Figure/FigS4.jpg]

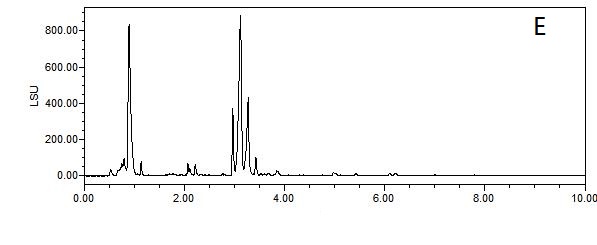

Supplement: Supplementary file 1 [file molecules-24-03269-s001.zip › Supplement/Supplementary Figure/FigS5.jpg]

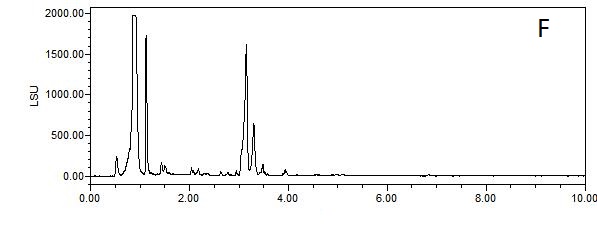

Supplement: Supplementary file 1 [file molecules-24-03269-s001.zip › Supplement/Supplementary Figure/FigS6.jpg]

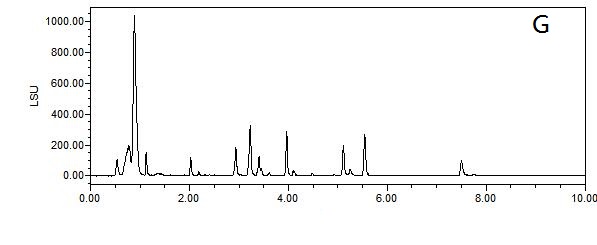

Supplement: Supplementary file 1 [file molecules-24-03269-s001.zip › Supplement/Supplementary Figure/FigS7.jpg]

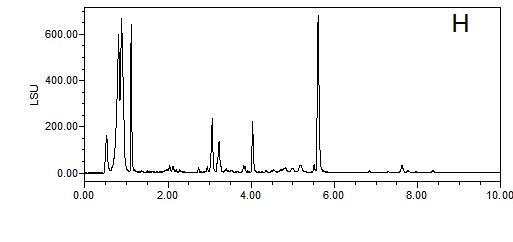

Supplement: Supplementary file 1 [file molecules-24-03269-s001.zip › Supplement/Supplementary Figure/FigS8.jpg]

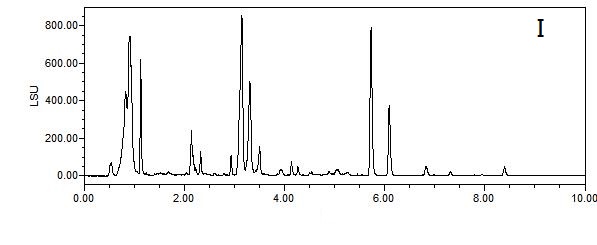

Supplement: Supplementary file 1 [file molecules-24-03269-s001.zip › Supplement/Supplementary Figure/FigS9.jpg]
